# *Burkholderia cepacia* in cystic fibrosis children and adolescents: overall survival and immune alterations

**DOI:** 10.3389/fcimb.2024.1374318

**Published:** 2024-07-01

**Authors:** Galina Shmarina, Daria Pukhalskaya, Vassiliy Shmarin, Sergey Semykin, Lusine Avakyan, Stanislav Krasovsky, Anastasia Goryainova, Svetlana Kostyuk, Rena Zinchenko, Nataliya Kashirskaya

**Affiliations:** ^1^ Research Centre for Medical Genetics, Moscow, Russia; ^2^ Peoples’ Friendship University of Russia (RUDN University), Moscow, Russia; ^3^ First Moscow State Medical University, Moscow, Russia; ^4^ Russian Clinical Children’s Hospital, a separate structural unit of the Russian National Research Medical University, Moscow, Russia; ^5^ Pulmonary Research Institute, Moscow, Russia

**Keywords:** inflammation, *Burkholderia cepacia* complex, IL-17F, TNFα, MMP, lymphocyte sensitivity to steroid suppression, glucose metabolism disturbances, cystic fibrosis

## Abstract

**Background:**

In current literature there are only scarce data on the host inflammatory response during *Burkholderia cepacia* complex (Bcc) persistence. The primary objective of the present research was to carry out cross-sectional analyses of biomarkers and evaluate disease progression in cystic fibrosis (CF) patients with chronic Bcc infection and pathogen-free ones. The secondary aim was to assess prospectively overall survival of the study participants during up to 8 years of follow-up.

**Methods:**

The study included 116 paediatric patients with CF; 47 CF patients were chronically infected with Bcc, and 69 individuals were Bcc free. Plasma and sputum biomarkers (neutrophil elastase, MMP-8, MMP-9, MMP-12, IL-2, IL-4, IL-6, IL-8, IL-10, IL-18, IL-22, IL-23, IL-17, IFN-γ, TGFβ_1_, TNF-α) were analysed using commercially available kits. Besides, inhibitory effect of dexamethasone on proliferative response of PHA-stimulated peripheral blood lymphocytes had been assessed.

**Results:**

Bcc infected patients did not differ from Bcc free ones in demographic and clinical parameters, but demonstrated an increased rate of glucose metabolism disturbances and survival disadvantage during prolong follow-up period. Biomarkers analyses revealed elevated TNF-α and reduced IL-17F levels in sputum samples of Bcc infected patients. These patients also demonstrated improvement of peripheral blood lymphocyte sensitivity to steroid treatment and reduction in plasma pro-inflammatory (IL-17F and IL-18) and anti-inflammatory (TGFβ1 and IL-10) cytokine concentrations.

**Conclusions:**

Reduction in IL-17F levels may have several important consequences including increase in steroid sensitivity and glycemic control disturbances. Further investigations are needed to clarify the role of IL-17 cytokines in CF complication development. Low plasma TGFβ1 and IL-10 levels in Bcc infected group may be a sign of subverted activity of regulatory T cells. Such immune alterations may be one of the factors contributing to the development of the *cepacia* syndrome.

## 1 Introduction

In 1947, an American plant pathologist Walter Burkholder first described *Pseudomonas cepacia* as a plant pathogen capable of causing onion rot (Burkholder, 1948). Later, additional *P. cepacia* isolates were detected and it became clear that this group of bacteria is occupying a wide array of ecological niches including rhizosphere of plants and freshwater environments (Vandamme and Dawyndt, 2011). With the advent of rRNA–DNA hybridization and subsequently the rRNA gene sequencing methods, taxonomists revised the initial classification and transferred *P. cepacia* and six other species of the so-called *Pseudomonas rRNA group II* to the new genus *Burkholderia* (Yabuuchi et al., 1992). In 1997, Peter Vandamm et al. showed that *Burkholderia cepacia* originally classified as single species included at least five different genomovars. Thus, the collective of the genomovars was named as the *B. cepacia complex* (*Bcc*) (Vandamme et al., 1997). To date, Bcc includes at least 24 phylogenetically related, but genetically different species (Tavares et al., 2020; Wang et al., 2020). Bcc bacteria can thrive in the diverse range of environments (such as pharmaceutical solutions, hospital equipment, industrial setting, shampoo, oil, fuel) and is capable of a variety of complex interactions (Mahenthiralingam et al., 2008; Tavares et al., 2020). Thus, several Bcc bacteria can degrade different xenobiotic compounds and organic pollutants, including constituents of crude oils, pesticides, phthalates, and solvents (Tavares et al., 2020). For example, *B. vietnamiensis* strain G4 is one of the most efficient degraders of trichloroethylene (O'Sullivan and Mahenthiralingam, 2005). *Bcc* bacteria can proliferate within the rhizosphere and promote the growth of many important crops such as peas (colonized by *B. ambifaria*), maize (*B. cenocepacia*), rice (*B. vietnamiensis*) and wheat (*B. cepacia and B. cenocepacia*), protecting seedling plants against other bacteria, protozoa, nematodes, and fungal diseases, such as root rot or seed-damaging infections. Some of Bcc species are also capable of N_2_ fixation, thereby contributing to plant growth (Parke and Gurian-Sherman, 2001; Tavares et al., 2020). Unfortunately, all these fascinated bacterial species can cause opportunistic infection in immunocompromised individuals, including cystic fibrosis (CF) patients and those with chronic granulomatous disease (CGD) (Greenberg et al., 2009; Coutinho et al., 2011).

Cystic fibrosis is an autosomal recessive disease associated with the mutation in CF transmembrane conductance regulator (*CFTR*) gene, that encodes a protein of c-AMP gated chloride channel. Impairment of ionic transport across the apical surface of epithelia leads to a high absorption of natrium and water by epithelial cells. The result of this process is increased viscosity of cellular secrets, which disturbs the function of respiratory system, pancreatic gland, hepatobiliary system, intestine and urogenital tract (Shteinberg et al., 2021). Mucociliary clearance impairment and CFTR deficiency related immune disturbances make respiratory tract an ideal environment for bacterial colonization (Ribeiro et al., 2023; Thornton and Parkins, 2023). Microbiological landscape in CF airways changes drastically through age and stage of disease. *Staphylococcus aureus* and *Haemophilus influenzae* dominate in early childhood and at early stages of CF lung disease. These bacteria “pave the way” for other microorganisms, such as *P. aeruginosa* and the *Burkholderia cepacia complex*, which prevail in the sputum samples in the later stages of the disease through adolescence and early adulthood (Ribeiro et al., 2023; Thornton and Parkins, 2023). Chronic lung inflammation is characterized by non-productive exuberant neutrophil infiltration and increased cytokine (TNF-α, IL-8, IL-17), neutrophil elastase, and MMPs production. Recurrent lung exacerbations lead to structural destruction of CF airways, respiratory tract obstruction and lung tissue remodeling. Progressive lung disease remains the leading cause of mortality in CF patients (Caverly et al., 2022; Ribeiro et al., 2023).

*Burkholderia* species are naturally resistant to many antibiotics (aminoglycosides, quinolones, β‐lactams, and host antimicrobial peptides including β‐defensins) making their eradication very difficult (Rhodes and Schweizer, 2016; Scoffone et al., 2017; Ganesh et al., 2020). Though only a small proportion of CF patients are infected with Bcc (1.0% in USA (Cystic Fibrosis Foundation Patient Registry, 2021) and 0.0-6.1% in European countries (Orenti et al., 2023), they represent a major concern, since individual outcomes are unpredictable and can vary from asymptomatic carriage to “*cepacia* syndrome” (Sfeir, 2018). This is an acute necrotizing pneumonia with an almost inevitable fatal outcome. *Cepacia* syndrome can be developed soon after the first acquisition of Bcc but it can also occur many years after the first organism isolation (Blackburn et al., 2004; Gilchrist et al., 2012). There is extensive literature on Bcc virulence factors including host response induction and mechanisms of drug resistance (Ganesan and Sajjan, 2012; Scoffone et al., 2017; Sfeir, 2018; Ganesh et al., 2020). However, most studies concerning host-pathogen interactions have been performed using cell cultures (Wright et al., 2011; Guadalupe Cabral et al., 2017; Dorrington et al., 2021) or animal models (mice, zebrafish) (Zgair, 2012; Mesureur et al., 2017; Loeven et al., 2021). At the same time, data on the inflammatory process in CF patients with chronic Bcc infection are largely missing in the literature. We found only a few references to cytokine levels in plasma and sputum samples of CF patients with chronic Bcc infection. These studies compared inflammatory markers of CF subjects with different microbiological characteristics and included a limited number of Bcc infected participants (n≤12) (Watt et al., 2005; Brazova et al., 2006; Hansen et al., 2010).

The primary objective of the present study was to carry out cross-sectional analyses of local and systemic biomarkers in Bcc infected CF patients (n=47) and in CF subjects who were Bcc free (n=69). In addition, the rate of non-pulmonary complications and concomitant diseases in two patient groups had been compared. The secondary aim was to assess prospectively overall survival of the study participants during up to 8 years of follow-up.

## 2 Methods

### 2.1 Study design

**Figure 1:**
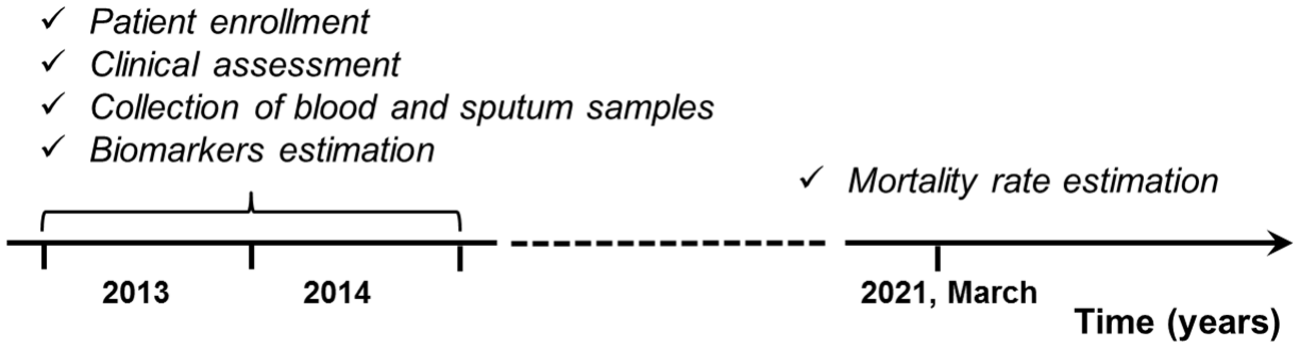
Study design.

There were 116 CF paediatric patients (age less than 18 years) who had attended the Cystic Fibrosis Department of the Federal State Budgetary Institution «Russian Paediatric Clinical Hospital» in Moscow between January 2013 and December 2014 enrolled in the study. Current clinical, microbiological and functional data were obtained. Blood and sputum samples of the patients had been collected and cross-sectional analyses of local and systemic biomarkers were performed. Then mortality rate had been prospectively evaluated during up to 8 years of follow-up period (**Figure 1**).

### 2.2 Patient assessment

CF was diagnosed by positive sweat test (chloride concentrations >60 mmol/L), typical clinical symptoms, and/or detection of CF causing variants. The *CFTR* genotype was determined in 111 patients: 44 individuals were homozygous for the *F508del*, 42 were heterozygous for the F508del, 25 subjects were carriers of other *CFTR* mutations and 5 had unknown/missing genotypes.

Spirometric tests were also carried out in children >6 years of age during periods of clinical stability. Spirometric data (forced vital capacity (FVC) and forced expiratory volume exhaled during the first second (FEV_1_), % predicted) were obtained from 110 of 116 patients. The limiting factors for spirometry performance were age less than 6 years old (4 patients), neurological problems (1 patient) and hemoptysis (1 patient). Respiratory microbial flora was determined by microscopy and culture of lower respiratory tract secretions or throat swabs performed at every routine visit to the CF Department. Bacterial identification and genotyping technology were conducted according to standardized protocols established for CF patients (Chernukha et al., 2014; Voronina et al., 2018). Chronic airway colonization was defined by the persistence of the pathogen in at least three airway samples for a period of at least 12 months.

According to European Cystic Fibrosis Society Standards (Smyth et al., 2014), cystic fibrosis-related liver disease was defined by at least one of the following criteria: 1) documented persistent increase in serum concentrations of the liver enzymes alanine aminotransferase (ALT, at least twice normal), aspartate transaminase (AST, at least 1.5 times normal), alkaline phosphatase (at least 1.5 times normal), or gamma-glutamyltransferase (GGT, at least 1.5 times normal); 2) persistent hepatomegaly (a percussed liver span greater than 1 SEM for age) for more than 6 months; 3) splenomegaly (a palpable spleen greater than 2.0 cm below the left costal margin); 4) abnormalities on ultrasound scan (liver with increased size, dishomogeneous echogenicity, or nodules with irregular margins; splenomegaly) for more than 6 months (Smyth et al., 2014).

Disturbances of glucose metabolism were assessed using the routine oral glucose tolerance test (OGTT) in accordance with European Cystic Fibrosis Society Standards (Smyth et al., 2014). All patients were clinically stable at the time of the OGTT, with no recent pulmonary exacerbations or symptoms suggestive of acute infection. Fasting plasma glucose (FPG) and 2-hour plasma glucose (2hPG) levels were measured after an oral glucose load of 1.75 g/kg of body weight (maximum 75g). FPG levels ≤5.5 mmol/L and 2hPG levels ≤7.8 mmol/L were considered to be consistent with normal glucose tolerance. Impaired glucose tolerance (IGT) was diagnosed if the 2hPG was from 7.9 to 11.0 mmol/L, and CF-related diabetes (CFRD) was diagnosed if the 2hPG was >11.0 mmol/L (Smyth et al., 2014).

All CF patients were treated with basic therapy following the European Cystic Fibrosis Society Standards (Smyth et al., 2014); treatment included mucolytics (dornase-alpha), multivitamins, high calorie diet, and microspheric pancreatic enzymes. In the case of acute pulmonary exacerbation, patients received antibacterial treatment which depended on the sputum microbiological analysis.

### 2.3 Blood collection and sputum processing

Blood and sputum collecting was performed during the years 2013-2014 (see **Figure 1**). At the time of blood and sputum sampling, all patients were clinically stable, without any recent pulmonary exacerbations or symptoms of acute infection. Venous blood was collected in EDTA tubes by venipuncture. The tubes were centrifuged at 400 × *g* for 10 min at 4°C to pellet the cells. Plasma was harvested, aliquoted and stored at –70°C for up to 3 months.

The sputum samples were placed in ice and delivered to the laboratory within 1 h. Each sputum sample was weighted, and double the weight of phosphate-buffered saline without Ca^2+^ and Mg^2+^ was added to the sputum specimen. The mixture was put on vortex for 40 s and then on the rocker for 30 min. Then sputum samples were filtered through 100 μm filters to get rid of mucus, and centrifuged at 400 x *g* for 10 min at 4°C to pellet the cells. The supernatants were collected, aliquoted, and stored at –70°C for up to 3 months. Protein concentrations in the samples were evaluated by Bradford’s method (Bradford, 1976).

### 2.4 Cytokine, adrenocorticotropic hormone and matrix metalloproteinase assessments

Cytokine and ACTH assessments were performed in accordance with the protocol of the research program “Immunological Monitoring of Cystic Fibrosis Patients”, developed by Research Centre for Medical Genetics since 1998. MMP-8 (Quantokine^®^ ELISA, R&DSystems), MMP-9 (Quantokine^®^ ELISA, R&DSystems), MMP-12 (Cloud-Clone Corp.) in sputum; IL-2 (High Sensitivity ELISA), IL-6 (High Sensitivity ELISA), TGFβ_1_, IL-10HS, IL-18, IL-22, IL-23 (Bender MedSystems GmbH; Vienna, Austria) in plasma; neutrophil elastase (Bender MedSystems GmbH; Vienna, Austria); TNF-α, IL-10, IFN-γ, IL-4, IL-8 (CYTOKINE; St. Petersburg, Russia), IL-17A and IL-17F (Bender MedSystems GmbH; Vienna, Austria) in plasma and sputum samples were analysed by ELISA technique with commercially available kits in accordance with the manufacturers’ instructions. Sputum cytokine levels were normalized to the protein content of each sample. Plasma ACTH was also measured using a commercially available kit (Diagnostic Systems Laboratories, Inc.; Webster, TX, USA). ELISA kits of the same lots were used for biomarker measurements.

### 2.5 Dexamethasone induced suppression of lymphocyte proliferation

Peripheral blood lymphocytes (PBL) were isolated from heparinazed peripheral blood by Ficoll-hypaque density gradient centrifugation. The cells were washed twice in phosphate-buffered saline without Ca^2+^ and Mg^2+^, and resuspended in RPMI-1640 medium supplemented with 10% heat-inactivated donor horse serum, 2x10^–3^ M HEPES, 2 mM L-glutamine, 2.8x10^–6^ M 2-mercaptoethanol, and 20 mg/ml gentamycin. The cells (5x10^4^ cells/well) were seeded in flat-bottomed 96-well plates and stimulated with PHA in the final concentration 5 mg/ml. Inhibition lymphocyte proliferation by dexametasone was evaluated at six different concentrations (10^–10^ to 10^–6^ M). Dexametasone was not added to the control wells containing a culture medium with or without PHA). The cells were incubated for 72 h at 37°C in humidified atmosphere containing 5% CO_2_. Four hours before the end of cultivation, each well was pulsed with 40 kBq of (^3^H)-thymidine (Isotope, Russia). The cells were harvested with a cell harvester and counted on a liquid scintillation counter. Triplicate wells of each concentration were assayed and the counts per minute (count/min) were averaged. Percentage inhibition was calculated by dividing the count/min in each inhibited sample by the count/min in the sample containing PHA only. The intensity of suppression was expressed as ED_50_. To evaluate individual susceptibility to glucocorticoids, the Δh parameters were calculated as described in our early studies (Pukhalsky et al., 1990, Pukhalsky et al., 1999; Shmarina et al., 2001). Previously the direct positive correlation between the level of PHA-induced lymphocyte proliferation and the inhibition degree of such stimulation by dexamethasone (ED_50_) has been shown. On the basis of this correlation the method of evaluation of individual susceptibility to the antiproliferative effect of glucocorticoids by Δh parameter calculation had been proposed (Pukhalsky et al., 1990).

Δh was calculated using formula:


$$\text{Δh}=\text{Y}–{\text{Y}}^{\prime },$$

where Y = lnED_50_, the experimental parameter; Y′ = lnED_50_′, the expected parameter; lnED_50_′= 0.447X – 4.399, X = ln ([count per min] in the samples treated with PHA only).

In this study we evaluated inhibitory effect of dexamethasone on proliferative response of PHA-stimulated lymphocytes obtained from 11 Bcc infected and 10 Bcc free CF subjects enrolled into the study. Their Δh values were compared with proper parameters of healthy volunteers and patients with chronic obstructive pulmonary disease (COPD). COPD group consisted of 47 patients (mean age, 48.4 ± 1.8 years; 27 males) who had attended the Clinical Department of Laboratory of Pulmonology, Moscow State University of Medicine and Dentistry named after A.I. Evdokimov, Moscow, Russia. Diagnosis of COPD confirmed by post-bronchodilator FEV_1_/EVC<0.7 during a stable state. The healthy group included 32 non-smoking volunteers (mean age, 28.2 ± 1.1 years; 15 males) with a negative history of respiratory disease or intercurrent illness.

### 2.6 Statistical analysis

Quantitative characteristics are presented in the form: mean ± standard error of the mean. Descriptive statistics of cytokines are expressed as the median (minimum value ÷ maximum value). Qualitative variables are expressed in their respective frequencies. Comparison of patient characteristics by groups was performed using the Mann–Whitney U-Test, Fisher’s exact test and the χ2 test. The odds ratio (OR) and 95% confidence interval (95% CI) were calculated. The Kaplan-Meier method was used to generate survival curves, and differences between the groups were assessed using a log-rank test. For univariate analysis and to estimate the independent effects of variables, a Cox proportional hazards model was used with 95% confidence intervals. Four independent categorical variables were analyzed via univariate Cox regression analysis: female sex, Bcc infection, glucose metabolism disturbance, low-dose prednisolone therapy. P-values less than 0.05 were considered statistically significant. Statistical analyses were performed using the statistical package SPSS, version 22.0 (IBM Corp.; Armonk, NY, USA) and STATISTICA 10 (StatSoftInc.; Tulsa, OK, USA).

## 3 Results

### 3.1 Bcc infected CF patients did not differ from non-infected ones in term of demographic and clinical data

Depending on microbiological status, all patients were divided into two groups: children who were chronically infected with Всс and those who were Bcc free. Demographic and clinical data are shown in **Table 1**. Bcc infected patients did not differ from Bcc free subjects in sex, age, and number of *F508del* mutation carriers. Similarly, clinical course of CF lung disease in Bcc infected children did not tend to be more severe than that in Bcc uninfected ones. Thus, mean values of FEV_1_, FVC and body mass index (BMI) did not differ between the patient groups. Bcc infected patients did not more frequently require prescribing of long-term low-dose prednisolone therapy (see **Table 1**). Nine patients with Bcc (18.4%) and 8 patients without the pathogen (11.6%) died during cross-sectional period of the study. Individual characteristics of dead patients are presented in **Supplementary Table 1**. The mean time from Bcc infection onset to death was 4.8 ± 0.5 years. Dead Bcc infected patients tended to be older than dead children from the Bcc free group (14.5 ± 1.3 *vs* 11.5 ± 1.3 years old, respectively, p=0.092) and had increased frequencies of CF-related complications such as diabetes and cirrhosis with portal hypertension (8/9 *vs* 2/8, respectively, p=0.013). In the same time all dead children from Bcc free group received oral steroids whereas there were 4 Bcc infected patients who were treated with prednisolone (p=0.020).

**Table 1:** Characteristics of the study subjects during cross-sectional period of the study.

	Patients		p
	with Bcc (n=47)	without Bcc (n=69)	
Age (years)	12.9 ± 0.5	12.2 ± 0.5	0.4477
Sex (Мale/Female)*	17/30	26/43	0.5134
BMI (kg/m^2^)	15.9 ± 0.4	15.8 ± 0.4	0.6834
*F508del* carriers*	34/44 (77.3%)	52/67(77.6%)	0.5719
Prednisolone treatment*	20/47 (42.6%)	31/69 (44.9%)	0.4757
FVC (% predicted)	80.9 ± 3.9	77.0 ± 3.3	0.2801
FEV_1_ (% predicted)	69.2 ± 4.4	68.4 ± 3.5	0.8860
Mortality rate*	9/49 (18.4%)	8/69 (11.6%)	0.2207

*The data were analyzed using Fisher exact test. Age, BMI, FVC and FEV1 values are shown as the mean ± standard error of the mean; the differences between the groups were assessed using Mann-Whitney U Test. BMI – body mass index; FVC – forced vital capacity (% of predicted values); FEV_1_ – forced expiratory volume exhaled during the first second, (% of predicted values).

### 3.2 Microbiological landscape in airways of Bcc infected/free patients

Airway microbial community has a great impact on CF lung disease progression. Therefore, the study of CF lung microbiology is as important as demographic and clinical data evaluation. Repeated microbiological studies revealed the presence of traditional CF pathogens (**Table 2**). The most common combinations were *В. cepacia* with *S. aureus* (16/47, or 34.0%) or *P. aeruginosa* with *S. aureus* in Bcc free group (43/69, or 62.3%).

**Table 2:** Microbiological characteristic of the study subjects during a cross-sectional period of the study.

	Patients		p*
	with Bcc (n=47)	without Bcc (n=69)	
*St. aureus*	**16/47 (34.0%)**	**43/69 (62.3%)**	**0.0024**
* *MRSA*	**0/47**	**6/69 (8.7%)**	**0.0404**
*P. aeruginosa*	**12/47 (25.5%)**	**48/69 (69.6%)**	**<0.001**
*Stenotrophomonas malt.*	2/47 (4.3%)	1/69 (1.5%)	0.3583
*Achromobacter xyl.*	**0/47**	**19/69 (27.5%)**	**<0.001**
Other **	15/47 (31.9%)	20/69 (29.0%)	0.4458

*- The results were analyzed using Fisher’s exact test;

**- Candida albicans, Streptococus viridans group, Acinetobacter sp., Ralstonia picettii, E. coli.

All statistically distinct parameters are highlighted in bold.

Twelve (25.5%) Bcc infected subjects and 48 (69.6%) Bcc free patients had chronic colonization with *P. aeruginosa* (p<0.001; **Table 2**). There were no cases of methicillin resistant *Staphylococcus aureus* (*MRSA)* infection among Bcc infected patients. In the same time Bcc free group included six patients with *MRSA* (p=0.0404). *Achromobacter xyl*. infection was revealed in 19 Bcc free subjects whereas nobody of Bcc-colonized patients were infected with the pathogen (p<0.001).

Most patients (45 of 47) were colonized with *B. cenocepacia*, the most abundant Bcc species which constituted 93.0% of all Bcc cases in Russia CF cohorts between 2012-2017 yrs (31). One patient (2%) had *B. contaminans*, the other one (2%) was infected with *B. vietnamiensis* (**Supplementary Figure 1**).

### 3.3 Frequency of CF complications and concomitant diseases during cross-sectional period of the study

CF complications and concomitant diseases contribute to lung disease progression, worsen the prognosis of the disease, and sometimes become a cause of death. Frequency of CF complications and concomitant diseases in the Bcc infected/free groups are presented in the **Table 3**. There were no significant differences in the frequencies of CF-related liver disease (including cirrhosis complicated with portal hypertension) between the Bcc infected and Bcc free patients. However, the patients colonized with Bcc were more likely than those in the Bcc free group to exhibit disturbances in glycemic control (X^2 ^= 6.25, p = 0.012; OR 3.34 (95% CI 1.36–8.19)). The risks for abnormal glucose metabolism among Bcc infected patients who received long-term low-dose prednisolone treatment or who had this therapy in their recent history, were further increased (X^2 ^= 5.30, p = 0.021; OR 4.17 (95% CI 1.19–14.54). There were no differences in number of patients receiving oral steroids between Bcc infected and Bcc free groups: 20/47 (42.6%) *vs* 31/69 (44.9%), respectively (see **Table 1**). However, 10 of 20 (50%) and 6 of 31 (19.4%) of the steroid-treated patients from Bcc infected and Bcc free groups, respectively, developed glucose metabolism disturbances.

**Table 3:** CF complications and concomitant diseases during cross-sectional period of the study.

	Patients		p
	with Bcc (n=47)	without Bcc (n=69)	
**CF-related liver disease (total)**	17/47 (36.2%)	23/69 (33.3%)	0.4521
*•* cirrhosis complicated with portal hypertension	6/47 (12.8%)	13/69 (18.8%)	0.2730
**Glucose metabolism disturbances (total)**	**17/47 (36.2%)**	**10/69 (14.5%)**	**0.0117**
• glucose tolerance impairment	8/47 (17.0%)	5/69 (7.3%)	*0.0914*
• CF-related diabetes	9/47 (19.2%)	5/69 (7.3%)	*0.0871*
• among patients with long term low-dose prednisolone therapy	**10/20 (50.0%)**	**6/31(19.4%)**	**0.0235**
**Atopic and inflammatory disorders (total**)	24/47 (51.1%)	32/69 (46.4%)	0.3795
*•* Drug intolerance	**24/47 (51.1%)**	**22/69 (31.9%)**	**0.0302**
*•* Other disorders (total)	**0/47**	**10/69 (14.5%)**	**0.0042**
*• allergic rhinitis*	0/47	2/69 (2.9%)	0.3517
*•bronchial asthma*	0/47	4/69 (5.8%)	0.1207
*• dermatitis*	0/47	3/69 (4.3%)	0.2067
*• arthritis*	0/47	2/69 (2.9%)	0.3517

The data were analyzed using Fisher exact test.

All statistically distinct parameters are highlighted in bold.

Atopic and inflammatory disorders were diagnosed in 24 of 47 (51.1%) patients with Bcc infection and in 32 of 69 (46.4%) Bcc free subjects. Drug intolerance episodes were more common in Bcc infected patients (24/47, 51.1%) than in Bcc free children (22/69, 31.9%; p=0.030). Also, remarkably, none of the patients with chronic Bcc infection developed or had recent history of arthritis and atopic diseases, but 10 Bcc free subjects were found to have concomitant inflammatory disorders including allergic rhinitis, bronchial asthma, dermatitis and arthritis as well (p=0.004; see **Table 3**).

### 3.4 Bcc infected CF patients demonstrated increase in TNFα and reduction in IL-17 F levels in their sputum samples

Sputum samples were obtained from 107 CF participants (**Table 4**). The patients with chronic Bcc colonization had higher concentrations of sputum TNFα compared to those of non-infected subjects (27.1 ± 9.2 and 6.1 ± 1.2 pg/mg protein, respectively, p=0.032). Besides, patients from Bcc infected group tended to show almost 2-fold increase in MMP-9 level in their sputum samples: 1124.8 ± 247.8 *vs* 573.5 ± 74.4 ng/mg protein in Bcc free group (p=0.056*)*. In the same time there was found a significant reduction in sputum IL-17F and clear trend to decline in sputum MMP12 in Bcc infected group compared to Bcc free one (7.1 ± 2.3 *vs* 23.1 ± 9.3 pg/mg protein, p=0.029 and 12.3 ± 3.9 *vs* 22.8 ± 4.5 ng/mg protein, respectively; p=0.074). Other biomarkers including neutrophil elastase, IL-8, IL-10, IFNγ, IL-4, MMP-8 were detected in the sputum samples, but no difference in concentrations was found when comparing the groups (see **Table 4**). The concentrations of IL-17A in the sputum samples of the most study participants were below detection limit (0.5 pg/ml).

**Table 4:** Sputum biomarkers.

*In situ* biomarkers,/mg protein		Patients		p
	*Detection limit*	with Bcc (n=44)	without Bcc (n=63)	
Elastase,	*1.98 pg/ml*	36.9 0 ÷;397.9	32.0 0 ÷;507.8	*0.5381*
IL-8,	*10 pg/ml*	532.5 62.7÷;8186.1	859.7 34.5÷;2955.3	*0.7916*
IL-17F,	*3.3 pg/ml*	**2.9** **0 ÷;55.8**	**5.9** **1.0 ÷; 305.2**	***0.0288***
TNFα,	*1 pg/ml*	**11.6** **1.2÷;155.7**	**4.4** **0.7÷;22.5**	***0.0317***
IL-10,	*5 pg/ml*	3.7 0÷;52.9	2.3 0÷;357.4	*0.3253*
IFNγ,	*20 pg/ml*	3.9 0 ÷;265.8	5.8 0 ÷;34.8	*0.3755*
IL-4,	*2 pg/ml*	1.3 0÷;282.4	1.4 0÷;130.5	*0.8903*
MMP-8,	*20 pg/ml*	338.9 232.5÷;1171.9	289.2 68.2÷;613.0	*0.2868*
MMP-9,	*0.156 ng/ml*	*1161.1* *213.5÷;2566.5*	*672.7* *97.3÷;1002.9*	*0.0564*
MMP-12,	*0.119 ng/ml*	*7.5* *1.8÷;44.1*	*18.7* *2.9÷;64.5*	*0.0739*

Data are presented as median values (minimum value ÷; maximum value). The results were analyzed using Mann-Whitney U test.

All statistically distinct parameters are highlighted in bold.

### 3.5 Bcc infected patients showed a reduction in both pro- and anti-inflammatory cytokine levels in their plasma samples

Plasma biomarkers were assessed for the 46 Bcc infected patients and 69 Bcc free persons. There were some differences in the biomarker patterns of the groups (**Table 5**). There were many *in vitro* and *ex vivo* studies showing exaggerated proinflammatory cytokines production by wild and CFTR-deficient cells treated with Bcc strains or Bcc LPS (Zughaier et al., 1999; Ganesan and Sajjan, 2012). Strikingly, in our study Bcc infected children showed a marked reduction in both pro- and anti-inflammatory cytokine levels in their plasma samples (**Table 5**). Thus, mean plasma IL-17F and IL-18 concentrations were, respectively, 34.3 ± 1.9 and 132.6 ± 36.4 pg/ml in Bcc infected group *vs* 50.8 ± 3.8 pg/ml and 415.8 ± 117.1 pg/ml in Bcc free patients (both p<0.031). Mean values for plasma IL-10 and TGF-β1 levels had amounted, respectively, 7.5 ± 1.5 pg/ml and 10.1 ± 1.1 ng/ml in Bcc infected group *vs* 34.9 ± 9.6 pg/ml and *vs* 14.5 ± 1.0 ng/ml in Bcc free group (both p<0.01).

**Table 5:** Plasma biomarkers.

Plasma biomarkers,/ml		Patients		p
	*Detection limit*	with Bcc (n=46)	without Bcc (n=69)	
Elastase,	*1.98 pg/ml*	81.4 26.1÷;815.3	68.6 28.9÷;164.8	*0.3599*
IL-8,	*10 pg/ml*	515.2 76.0÷;3424.1	366.5 36.8÷;8580.9	*0.4217*
TNFα,	*1 pg/ml*	3.8 1.5÷;7.4	4.6 1.4÷;25.2	*0.8481*
IL-10,	*5 pg/ml*	**5.0** **0÷;44.2**	**7.1** **0÷;598.5**	***0.0007***
IL-10 HS,	*0.05 pg/ml*	**0.84** **0.20÷;3.4**	**1.49** **0.18÷;30.0**	***0.0184***
IFNγ,	*20 pg/ml*	102.7 30.9÷;1840.4	105.7 29.2÷;2180.3	*0.3142*
IL-4,	*2 pg/ml*	*7.0* *0 ÷; 232.2*	*8.4* *0 ÷; 393.4*	*0.0883*
IL-2 HS,	*0,40 pg/ml*	0.92 0 ÷; 2.1	0.94 0 ÷; 1.9	*0.6094*
IL-6 HS,	*0,03 pg/ml*	0.78 0 ÷; 9.35	2.19 0 ÷; 11.2	*0.5197*
IL-17F,	*3.3 pg/ml*	**32.2** **9.7 ÷; 60.9**	**43.8** **19.8 ÷; 107.9**	***0.0053***
IL-18,	*9 pg/ml*	**106.7** **0 ÷; 332.7**	**296.8** **0 ÷; 2885.4**	***0.0309***
IL-22,	*5 pg/ml*	25.1 21.5 ÷; 103.2	24.8 20.3 ÷; 36.9	*0.9483*
IL-23,	*4 pg/ml*	18.1 15.6 ÷; 21.9	16.7 15.9 ÷; 22.5	*0.2116*
TGF-β1,	*0.0086 ng/ml*	***8.8*** ***0 ÷; 29.6***	***13.1*** ***0 ÷; 53.0***	***0.0081***
ACTH,	*0.22 pg/ml*	13.7 2.3 ÷; 32.6	11.0 0 ÷; 91.6	0.1376

Data are presented as median values (minimum value ÷; maximum value).

The results were analyzed using the Mann-Whitney U test.

All statistically distinct parameters are highlighted in bold.

There were no significant differences between the groups in plasma levels of neutrophil elastase, IL-8, TNFα, IL-10, IFNγ, IL-2, IL-4, IL-6, IL-22, IL-23, and ACTH levels (see **Table 5**). The concentrations of IL-17A and TNFα in the plasma samples of the most study participants were below detection limit (0.5 pg/ml and 1.0 pg/ml, respectively).

### 3.6 Peripheral blood lymphocyte susceptibility to steroid suppression

**Figure 2:**
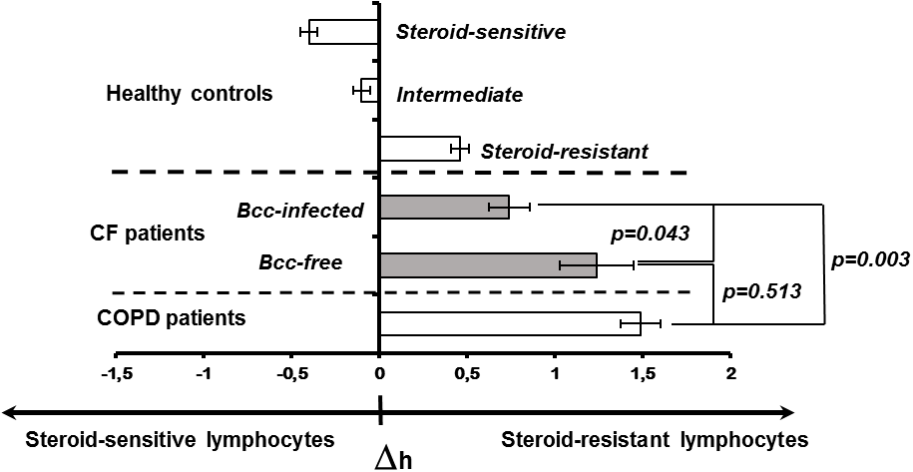
Peripheral blood lymphocyte susceptibility to steroid suppression. Inhibition degree of PHA-induced lymphocyte proliferation by different concentrations of dexamethasone was evaluated. Patients and healthy subjects were classified as steroid resistant if their Δh-parameters > 0 and steroid sensitive if their Δh-parameter > 0. The cell sensitivity is presented as the mean (± m) of the Δh value. The results were analyzed using the Mann-Whitney U test.

Decreased cytokine concentrations in plasma samples of Bcc infected patients suggest the reduction in systemic inflammatory response. Since PBL susceptibility to glucocorticoids is strongly correlated with the severity of systemic inflammation (Shmarina et al., 2001; De et al., 2002; Quax et al., 2013), we evaluated inhibitory effect of dexamethasone on proliferative response of PHA-stimulated lymphocytes obtained from Bcc infected (n=11) and Bcc free (n=10) patients. Nobody of the patients received long-term low-dose prednisolone treatment or had this therapy in the recent history. The data are presented in **Figure 2** and **Supplementary Table 2**.

There were no significant differences in the levels of PBL proliferative response between the patient groups. In the same time, the mean ED50 value of dexamethasone in Bcc infected patients was about twice lower than that in Bcc free group (31.6 ± 11.2 *vs* 67.0 ± 28.8 µM, respectively), but this difference did not reach statistical significance (p=0.183).

To evaluate individual susceptibility to glucocorticoids, the Δh-parameters were accounted as described in ‘Material and Methods’. **Figure 2** provides Δh values for healthy subjects, CF children and patients with severe COPD. Since Δh-parameters are not widely used in laboratory practice, the presented data can be a good illustration for changes of Δh values in health and disease. Besides, there are phenotypic similarities between COPD and CF pulmonary disease including airway surface liquid dehydration, mucus hypersecretion, pulmonary microbiome composition, as well as small airway disease with neutrophilic inflammation and lung remodelling (Mall et al., 2023; Miravitlles et al., 2024). Recent studies revealed acquired CFTR impairment in airways, sweat glands and intestines of COPD patients (Mall et al., 2023; Miravitlles et al., 2024). Similar to CF subjects with mutated *CFTR*, COPD patients demonstrated increased chloride concentrations in their sweat samples and elevated Beclin 1 level in peripheral blood (Raju et al., 2013; Schlemmer et al., 2018). The main reasons of acquired CFTR dysfunction include smoking, reactive oxygen species, inflammation and bacterial byproducts (Mall et al., 2023; Miravitlles et al., 2024). Local and systemic consequences of impaired CFTR expression and function in COPD have been discussed in comprehensive review by Miravitlles et al. Over the last decade, clinical studies of CFTR potentiators (ivacaftor and icenticaftor), being created to recover mutant protein activity in CF subjects with specific mutations, have been conducted in COPD patients (Solomon et al., 2016; Martinez et al., 2023).

There were statistically significant differences between the patient groups (CF, COPD) and steroid-sensitive, intermediate as well as steroid-resistant healthy controls (all p<0.035). However, CF subjects with chronic Bcc colonization were more steroid-sensitive then Bcc free CF children and COPD patients (both p<0.044). Thus, mean values for Δh-parameters in Bcc infected group had amounted 0.74 ± 0.12 abs. units *vs* 1.24 ± 0.21 (in Bcc free group) and 1.49 ± 0.11 abs. units (in COPD group). The values of Δh-parameters in the Bcc free CF patients and COPD subjects were statistically equivalent (p = 0.513).

Thus, during cross-sectional period of the study we revealed neither significant lung function failure nor survival disadvantage in Bcc infected patient group. Besides, these patients demonstrated the reduction in systemic inflammatory response in comparison with Bcc free CF subjects. To clarify the data obtained we continued to follow-up microbiological status and lifespan of the examined patients.

### 3.7 Outcomes: survival and Bcc colonization longevity

**Figure 3:**
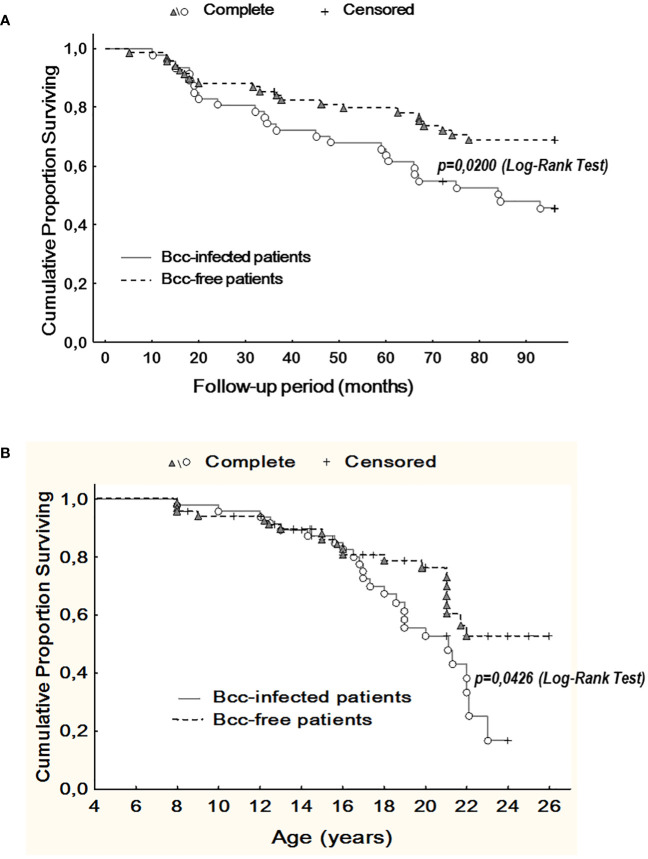
Kaplan-Meier survival analysis of Bcc infected and Bcc free patients. **(A)** Cumulative proportion surviving *vs* Follow-up period; **(B)** Cumulative proportion surviving *vs* Age of the patients. The plots indicate that chronic Bcc-colonization is associated with a worse outcome. The end points were considered as deaths. Bcc – *Burkholderia cepacia* complex.

During up to 8 years follow up, we observed 46 deaths. Of these, 25 deaths occurred in Bcc infected group and 21 deaths were documented in Bcc free cohort (**Supplementary Table 3**). Thereafter, survival rates in the patient groups were 46.8% and 69.6%, respectively (X^2^ = 6.05, p = 0.0139; OR 2.6 (95% CI 1.2–5.6)). During follow up period, patients infected with Bcc survived a median of 84.5 months, whereas there were insufficient death numbers in the Bcc free group to define a median. Kaplan-Meier survival analyses showed that Bcc infected patients had decreased overall survival compared with Bcc free ones (p=0.020, Log Rank test; **Figure 3A**).

Similarly, in Bcc free group, it was impossible to evaluate median survival time in years due to insufficient death numbers. In Bcc infected group median survival time in years was 21.1. Kaplan-Meier survival analysis demonstrated that Bcc-infected patients had a worse prognosis in overall survival than Bcc free CF subjects (p=0.043, Log Rank test; **Figure 3B**).

The Cox proportional hazards model was constructed to confirm the Kaplan-Meier survival analysis and determine the other variables that were associated with outcome (**Table 6**). Besides chronic Bcc colonization, glucose metabolism disturbance and low-dose long-term prednisolone therapy were also significantly associated with increased hazard ratios.

**Table 6:** Cox proportion hazards model for studied patients.

Variable	Hazard Ratio	95% CI	p value
	**Follow-up period (n=116)**		
Bcc infection*	**1.98**	**1.11 – 3.54**	**0.021**
Glucose metabolism disturbance**	**2.82**	**1.56 – 5.12**	**0.001**
Low-dose prednisolone therapy***	**2.65**	**1.46 – 4.79**	**0.001**
Sex, female****	1.06	0.58 – 1.93	0.855
	**Age (n=116)**		
Bcc infection	**1.80**	**1.01 – 3.23**	**0.047**
Glucose metabolism disturbance	**2.10**	**1.16 – 3.79**	**0.014**
Low-dose prednisolone therapy	**3.16**	**1.74 – 5.75**	**<0.0001**
Sex, female	1.26	0.69 – 2.30	0.448

*Hazard ratio is relative to non-Bcc infection.

**Hazard ratio is relative to normal glucose metabolism.

*** Hazard ratio is relative to absence of low-dose long-term prednisolone therapy in the recent history.

**** Hazard ratio is relative to men. All statistically distinct parameters are highlighted in bold

It was notably the mean age of Bcc acquisition in infected group was 8.9 ± 0.5 years. At the time of Bcc onset the survivors were about 2 years younger than dead patients: 7.9 ± 0.6 and 9.8 ± 0.7 years, respectively (p=0.0498; **Supplementary Table 4**). The mean value of Bcc colonization longevity was reached 9.2 ± 0.5 years. In the subgroup of survived Bcc subjects this parameter was 11.3 ± 0.3 years, in the cohort of dead Bcc patients – 7.4 ± 0.6 years. These data are in line with the recent study by Krasovsky et al. that included 138 Bcc infected and 419 Bcc free adult CF patients (Krasovskiy et al., 2019).

The microbiological follow-up did not reveal cases of chronic or intermittent Bcc colonization among uninfected patients.

## 4 Discussion

The number of CF patients with chronic Bcc infection is small (3.5% in worldwide) and continues to decline due to preventive measures and modern therapies (Sousa et al., 2021). Since chronic Bcc infection is much more common in adult patients, the current literature provides data only for adults or mixed patient populations (children and adults) (Folescu et al., 2015; Zlosnik et al., 2015; Granchelli et al., 2018; Krasovskiy et al., 2019; Somayaji et al., 2020). Thus, our study is pretty unique one, as it includes CF children and adolescents with chronic Bcc colonization. As in the other studies (Jones et al., 2004; Brazova et al., 2006; Krasovskiy et al., 2019; Somayaji et al., 2020), immunological parameters and survival of Всс infected patients have been compared with the non-colonized CF peers who had the same stage of the lung disease. Most of the Bcc free patients were chronically infected with *P. aeruginosa*, a minority has recurrent lung exacerbations related to chronic MRSA, *Stenotrophomonas maltophilia*, *Achromobacter xylosoxidans* infections.

The study consists of two parts: cross-sectional analyses of demographic, microbiological and immunological characteristics of Bcc free/infected CF children and prospective assessment of overall survival of the study participants. Cross-sectional period of this study had been performed in years 2013-2014 when there were limited data on the survival, outcomes and inflammatory response severity even in patient cohorts infected with specific Bcc species nothing to say about patient cohorts infected with *B. cenocepacia* specific strains. Historically, any *B. cenocepacia* infection was considered to be connected with rapid decrease of lung function, weight loss, exaggerated inflammatory response and greater risk of death or transplantation (Jones et al., 2004; Drevinek and Mahenthiralingam, 2010; Folescu et al., 2015; Zlosnik et al., 2015; Somayaji et al., 2020). This opinion was mainly based on clinical data of patients infected with clones of epidemic ET-12 lineage, a highly transmissible *B. cenocepacia* species which causes aggressive inflammatory response, resulting in progressive lung function failure and high mortality rate (Jones et al., 2004; Drevinek and Mahenthiralingam, 2010; Somayaji et al., 2020). In addition, the majority of *in vivo* and *in vitro* studies on the molecular pathogenesis of *B. cenocepacia* have used ET-12 clone prototypic strains (for example, J2315, BC7 or k56-2) demonstrating extremely high severity and virulence of this Bcc species (Wright et al., 2011; Mesureur et al., 2017; Dorrington et al., 2021; Loeven et al., 2021). In our study nearly all patients (45 of 47) of Bcc group were infected with *B.cenocepacia*. Median colonization longevity at the moment of biomarkers assessment was 4.5 (1.0 ÷ 8.0) years (see **Supplementary Table 4**). However our data indicate that Bcc infected pediatric patients did not differ from Bcc free CF peers in terms of FVC, FEV_1_ and BMI values, mortality rate, and number of subjects who required prescribing of long-term low-dose prednisolone therapy (see **Table 1**). Moreover, Bcc infected patients demonstrated the signs of reduction in systemic inflammatory response, such as decreased plasma cytokine concentrations and improvement of PBL steroid-sensitivity (see **Table 5** and **Figure 2**). Furthermore, none of the infected patients developed or had recent history of inflammatory or atopic diseases (excluding multiple episodes of drug intolerance) whereas 10 Bcc free subjects had concomitant inflammatory disorders. In the same time, there was found an increased rate of glucose metabolism disturbances in Bcc infected group (see **Table 3**). These results may seem somewhat unusual. We could not explain the inconsistency of our data to the generally accepted point of view and continued to follow-up microbiological status and lifespan of the examined patients. Nobody of uninfected individuals had been colonized with Bcc during follow-up period. Survival rates in Bcc infected/free groups were 46.8% and 69.6%, respectively (p=0.020). These data are completely in line with the results of two early studies demonstrating a survival disadvantage of Bcc infected patients in comparison with uninfected ones during prolong follow-up periods (Frangolias et al., 1999; Jones et al., 2004). In the same time the authors did not find any difference in annual changes of weight and spirometry parameters between the patient groups. Similarly, there were no differences in baseline FEV_1_% or the rate of FEV_1_% decline between the groups with and without Bcc infection in the recent retrospective cohort study by Somayaji et al (Somayaji et al., 2020). The median time from Bcc acquiring to death or transplantation for *B. cenocepacia* (excluding ET-12) group was 6.24 (*7.0, in our study*) *vs* 9.42 years for those without Bcc infection. In the univariate model, *Burkholderia* infection was associated with an HR of 1.48; 95% CI: 1.00 –2.19 (*1.98; 95% CI: 1.11 – 3.54, in our study*) for death or transplantation compared to those with no infection. For the patients infected with ET-12 clones, median time from Bcc onset to death and HR were 1.95 years and 3.92 (95% CI: 2.25 – 6.81), respectively (Somayaji et al., 2020).

Repeated microbiological examinations demonstrated limiting acquisition or persistence of other infections in children with Bcc colonization. Thus, during the steady-state period, nobody of Bcc colonized patients were infected with MRSA or *Achromobacter xylosoxidans* whereas Bcc free group included 6 patients with MRSA and 19 subjects with *Achromobacter xylosoxidans* infections in their recent history (see **Table 2**). These data are consistent with findings of previous studies (Folescu et al., 2015; Granchelli et al., 2018). *Folescu et al.* (Folescu et al., 2015) carried out a retrospective study of 56 patients (0-36 years) who were categorized into three groups (I: Bcc free, II: intermittent Bcc colonization, III: chronic Bcc colonization). Similar to our data, chronic MRSA colonization was found in 7.7% in group I, 7.4% in group II and 0% in group III. Using the data of Cystic Fibrosis Foundation Patients Registry, 2003-2011, Granchelli et al (Granchelli et al., 2018). performed analysis of 538,458 sputum cultures of 28,042 CF patients aged six and older from 257 accredited US Care Centers and Affiliates. All participants had sputum cultures for at least two consecutive years for MSSA, MRSA, *Pseudomonas aeruginosa*, *Burkholderia cepacia* complex, *Stenotrophomonas maltophilia*, *Achromobacter xylosoxidans*, *Candida* and *Aspergillus* species. This study showed that Bcc-colonization is associated with lower odds for concurrent and subsequent MRSA and/or *Achromobacter xylosoxidans* acquisition and persistence. In addition, there were strong negative associations between Bcc and MSSA as well as between Bcc and *P.aeruginosa* infections (Granchelli et al., 2018). In agreement, in our study Bcc free group included 43 (62.3%) patients with chronic MSSA and 48 (69.6%) patients with chronic *P.aeruginosa* colonization, whereas in Bcc infected group these parameters were 16 (34.0%) and 12 (25.5%), respectively. It is widely known that decrease in airway microbiome diversity in CF patients is associated with severe lung function failure and poor prognosis (Granchelli et al., 2018; Thornton and Parkins, 2023). Indeed, repeated courses of antibiotic therapy for recurrent lung exacerbations inevitably reduce variety of CF microbial community. Eventually, patients with advanced lung disease usually have poor microbiological landscape with a predominance of 1-2 typical CF pathogens (Thornton and Parkins, 2023). In CF, co-colonization with several pathogens has its benefits and drawbacks. Thus, co-colonization can promote antibiotic resistance development and subvert the host immune response (Limoli and Hoffman, 2019; Oliveira et al., 2023). In addition, co-occurring species maintain virulence factor production resulting in development of exuberant inflammatory response and, as a consequence, more rapid lung tissue destruction (Bottery et al., 2024). In such a situation, lung inflammation caused by intermittent infection with highly virulent strain of a new pathogen can be easily stopped due to already activated anti-inflammatory mechanisms. At the absence of competing species, the dominant pathogen evolves under pressure of the host immune system towards the decrease or loss of virulence factor production (Thornton and Parkins, 2023; Bottery et al., 2024). This pathoadaptation mitigates systemic inflammation, leading to decrease of both pro- and anti-inflammatory cytokine levels and increases lymphocyte sensitivity to steroid suppression (see **Table 5** and **Figure 2**). The destruction of lung tissue is slowed down. However, any accidental infection with a new highly virulent germ can break down peripheral tolerance to the dominant pathogen and trigger uncontrolled inflammatory reaction, quickly leading to death, as it occurs with *cepacia* syndrome (Blackburn et al., 2004; Gilchrist et al., 2012; Sfeir, 2018). It should be noted that the adapted poorly virulent pathogens are highly resistant to antibiotics and vaccination, and their complete eradication is impossible (Wang et al., 2020; Sousa et al., 2021).

*In situ* biomarkers assessment revealed decreased IL-17F and elevated TNFα levels in sputum samples of Bcc infected patients compared to those of uninfected ones. Besides, patients with Bcc tended to show an increase in sputum MMP-9 and reduction in MMP-12 concentrations (see **Table 4**). It is known, that both important opportunistic CF pathogens *B. cenocepacia* and *P. aeruginosa* induce NF-κB activation triggering TNFα and MMPs (including MMP-9 and MMP-12) expression in epithelial and immune cells (Zughaier et al., 1999; Bamford et al., 2007; McKeon et al., 2010; Wright et al., 2011; Park et al., 2015; Hendrix and Kheradmand, 2017). However, *in vitro* experiments had shown that alive Bcc strains and/or Bcc LPS elicit up to 25 times more TNF-α compared to strains and/or LPS of other CF pathogens including *P. aeruginosa* (McKeon et al., 2010; Cabral-Pacheco et al., 2020). It has been noted that unlike our study, Hansen et al. did not find any differences in TNFα levels in sputum samples of CF patients with Bcc (n=11) and *P. aeruginosa* (n=21) infections. However, the patients in this study were older (mean age, 29.5 and 28.5 years, respectively). In addition, the authors did not normalize TNF-α concentrations to the protein or urea content of each sputum sample (Hansen et al., 2010).

Several authors had reported a TNF-mediated induction of MMP-9 synthesis and release by non-CF immune and epithelial cells (Richardson, 2010; Cabral-Pacheco et al., 2020). Beeh et al. had shown that MMP-9 positively correlated with TNF-α in the sputum samples of lung transplant recipients and COPD patients, suggesting a regulatory role of TNF-α on MMP-9 expression and activation (Beeh et al., 2001, Beeh et al., 2003). In accordance with the above, in the study by Wright et al. *B. cenocepacia* strain BC7 (but not *B. multivorans* strain LMG13010, or *P. aeruginosa* strain PAO1) dramatically increased MMP-9 expression and activity in both CF and non-CF lung epithelial cells (Wright et al., 2011). The activation of MMP-9 correlated with an impairment of wound repair in confluent monolayers of lung epithelial cells. Specific inhibition of MMP-9 in medium from cells exposed to *B. cenocepacia* completely reversed this negative effect (Wright et al., 2011). Several studies have investigated the link between MMP-9 and CF lung disease severity. BAL fluid MMP-9 levels are elevated and negatively correlated with lung function, as measured by FEV_1_, in adults and children with CF (Sagel et al., 2005; Hilliard et al., 2007; Devereux et al., 2014; Hendrix and Kheradmand, 2017).

Our study is believed to be the first one reported sputum MMP-9 and MMP-12 levels in CF patients with chronic Bcc colonization. MMP-9 possessing high gelatinolytic activity can cleave collagen before prolyl endopeptidase further degrades the gelatin fragments to form neutrophil chemoattractant proline–glycine–proline (PGP), which contributes to the chronic neutrophilic inflammation in CF patients (Gaggar et al., 2008). In the same time, *in vivo* study by Hong et al. had shown that MMP-9 specifically inactivated the cytokine IL-17А, thereby terminating neutrophilia in lungs of mice with acute S. pneumoniae infection (Hong et al., 2011). MMP-12, a potent elastin-degrading proteinase, is secreted by lung macrophages (Shan et al., 2009). Previous studies had demonstrated that elevated levels of MMP-12 in the sputum were associated with emphysema severity in COPD and asthma patients. MMP-12 activity was found to be elevated on the surface of airway macrophages in bronchoalveolar lavage from young CF children with early lung disease. In addition, SNP in the MMP-12 promoter (rs2276109) and a tightly linked SNP (rs737693) were seemed to be positively associated with severity of CF lung disease (Trojanek et al., 2014). We did not find any data concerning effects of *B. cenocepacia* on MMP-12 expression and activity. In the same time *in vitro* investigation demonstrated that infection with *P. aeruginosa* strain K induced NF-κB mediated MMP-12 expression in human airway epithelial H292 cells (Park et al., 2015). Study by Shan et al. had shown that IL-17A, the canonical Th17 cell cytokine, enhanced secretion of MMP-12 from lung macrophages (Shan et al., 2009). So, specific immunological *in situ* profiles of Bcc infected group with increased TNF-α/MMP-9 and Bcc free group with elevated IL-17F/MMP-12 (see **Table 4**) are completely in line with the data of the recent studies.

IL-17A and IL-17 F are the most studied members of IL-17 cytokines family. They share more than 50% structural homology, form heterodimers and have two common receptors: IL-17RA and IL-17RC. Both cytokines possess similar pro-inflammatory activity, though IL-17A is the more potent than IL-17F (McGeachy et al., 2019). Dual IL-17A and IL-17F blockade in Th17 supernatants was more effective at reducing the secretion of pro-inflammatory mediators than blockade of IL-17A alone (Glatt et al., 2018). Recent clinical studies have shown that therapy with bimekizumab, a dual IL-17A and IL-17F neutralizing antibody, resulted to pronounced clinical responses in patients with psoriasis (Oliver et al., 2022) and psoriatic arthritis (McInnes et al., 2023). IL-17 cytokine family members and their role in promoting inflammation have been considered in several comprehensive reviews (Gurczynski and Moore, 2018; Brevi et al., 2020; McInnes et al., 2023). Analysis of mouse studies suggests that robust IL-17 cytokine production plays an ambivalent role in CF lung disease progression. On the one hand, proper IL-17 signaling is crucial in host defense against Gram-negative bacterial infections, protecting against chronic colonization and death (Bayes et al., 2016). On the other hand, exuberant IL-17 cytokine production can contribute to tissue damage through excessive neutrophil accumulation and induction of matrix metalloproteinases (Hsu et al., 2016). Indeed, higher percentages of Th17 cells in the blood were strongly associated with poor lung function (FEV1% predicted) in CF patients (Mulcahy et al., 2015).

Several studies have analyzed the expression of IL-17 cytokines in blood and sputum samples obtained from CF patients. Early study by McAllister et al. showed elevated levels of IL-17A and IL-17F in the sputum of CF patients who were colonized with *Pseudomonas aeruginosa* at the time of pulmonary exacerbation (McAllister et al., 2005), with IL-17F concentrations being higher. The cytokines demonstrated similar dynamics during exacerbation treatment, with significant reduction at day 20 of antibiotic therapy. Two other studies showed increased IL-17 mRNA in sputum (Decraene et al., 2010) and T-lymphocytes (Tiringer et al., 2013) obtained from *P. aeruginosa* infected patients. In contrast, the recent study by Oshalim et al. demonstrated that patients infected with typical bacterial CF pathogens had lower IL-17 A and IL-17 F levels in their sputa than CF subjects with only ordinary respiratory flora or non-CF patients with sputum production due to a mild acute respiratory infection (Oshalim et al., 2020). Besides, sputum IL-17A levels in CF adults and subgroup with *P. aeruginosa* infection were lower than those in CF children and *P. aeruginosa* non-infected subgroup. Similarly, decreased levels of IL-17 transcripts were found in PBL of *P. aeruginosa* positive patients compared to those of uninfected CF subjects and non-CF controls (Ghorban Movahed et al., 2022). Unfortunately, the levels of IL-17A both in sputum and plasma in most participants of our study, were below the sensitivity limit of the method. However, we also performed IL-17F measurements and found reduced concentrations of the cytokine both in plasma and in sputum samples of patients with Bcc infection compared to Bcc-free group. Our results and the data of others (Oshalim et al., 2020; Ghorban Movahed et al., 2022) suggest IL-17 cytokine reduction (congenital, age-related and/or pioneer CF species induced) can predispose CF lungs to future chronic Bcc infection. This assumption is fully consistent with the data of early *in vitro* study comparing the IL-17 expressions by PBL obtained from healthy controls and patients with diabetes mellitus, the most common risk factor for melioidosis (caused by Bcc related species *B. pseudomallei*). The results clearly demonstrated that the PBL from diabetic patients, stimulated with PHA (T-cell mitogen) as well as with *B. pseudomallei* and *B. thailandensis*, expressed significantly lower IL-17 mRNA and protein levels than those from healthy donors (Pongcharoen et al., 2008). Notably, we found increased frequency of glycemic control disturbances in Bcc-infected group (see **Table 3**).

A decrease in the level of IL-17F in Bcc-infected group can also have some other important consequences. The first one is the absence of concomitant inflammatory disorders (excepted drug intolerance episodes) in the recent history of the patients from Bcc infected group (see **Table 3**). These data are consistent with past researches showing the role of IL-17 cytokines in development of inflammatory disorders including allergic rhinitis (Gu et al., 2017), bronchial asthma (Sorbello et al., 2015), dermatitis (Sugaya, 2020) and arthritis (Miossec, 2021) as well. The second consequence of IL-17F reduction is improvement of PBL steroid-sensitivity (see **Supplementary Table 2**, **Figure 2**). Indeed, IL-17 cytokines affect the steroid responsiveness via up-regulation of glucocorticoid receptor (GR) β. This dominant negative GR isoform promotes steroid resistance through GRα-dependent mechanism by forming a non-transactivating heterodimer with GRα thereby impairing GRα-mediated activities (Ramos-Ramírez and Tliba, 2021). There are several *in vitro* studies suggesting GRβ up-regulation and steroid resistance induction in PBL, adipocytes and fibroblasts stimulated with IL-17 cytokines (Vazquez-Tello et al., 2013; Al Heialy et al., 2020; Lo et al., 2022). GRβ is associated with corticosteroid-insensitive asthma and idiopathic pulmonary fibrosis (IPF) (Al Heialy et al., 2020; Lo et al., 2022). Recent study by Al Heialy et al. showed that asthmatic patients with elevated serum IL-17F (but not IL-17A) had decreased serum GRα/GRβ ratios, and consequently the patients with reduced serum IL-17F exhibited high serum GRα/GRβ values (Al Heialy et al., 2020). Immunohistochemical staining of lung specimens from subjects with interstitial lung diseases (cryptogenic organizing pneumonia, sarcoidosis and IPF) revealed greater percentage of IL-17+ cells, stronger IL-17 and GRβ expression and higher GRβ/GRα ratio in IPF group, characterized by the poor responses to corticosteroid treatment. IL-17 expression was positively associated with GRβ and GRβ/GRα ratio in specimens obtained from all patient’s groups (Lo et al., 2022). IL-17 deficiency may be also related to increased rate of glucose metabolism disturbances in Bcc infected group. *In vivo* and *in vitro* studies suggest that IL-17 cytokines contribute significantly to the homeostatic regulation of glucose metabolism (Zúñiga et al., 2010; Bechara et al., 2021). *In vitro*, IL-17 inhibited insulin-induced glucose uptake by adipocytes (Zúñiga et al., 2010). *In vivo*, IL-17 deficiency enhanced glucose tolerance and insulin sensitivity in young mice, but simultaneously promoted adipocyte differentiation, leading to the increased accumulation of adipose tissue mass, the onset of obesity and the metabolic syndrome development. Even lean IL-17 deficient mice have found to demonstrate modest fasting hyperglycemia and reduced basal insulin levels suggesting that control of basal insulin secretion may also be affected (Zúñiga et al., 2010). In CF, insulin production is initially compromised due to basic defect. Besides, repeated episodes of lung disease exacerbation as well as high calorie diet recommended by European Standards additionally predispose CF subject to glycemic control disturbances. In this context, IL-17 deficiency related glucose metabolism abnormalities may increase risks of developing the glycemic control disturbances, the phenomenon we have found in Bcc-infected group. No wonder, the risks for abnormal glucose metabolism were further increased among Bcc-infected patients who received long-term low-dose prednisolone treatment or who had this therapy in their recent history (see **Table 3**). There are several other studies suggesting prevalence of CFRD and other disturbances of glycemic control in CF children and adults with chronic Bcc colonization (Sterescu et al., 2010; Granchelli et al., 2018; Olesen et al., 2020).

Besides reduced IL-17F levels, we also found decrease in pro-inflammatory IL-18 as well as anti-inflammatory TGFβ1 and IL-10 in plasma samples of Bcc infected group. Similarly, Brazova et al. found the minimal TGFβ1 production by the stimulated whole blood cell cultures of Bcc infected CF patients in comparison to groups of patients with *P. aeruginosa* or other CF pathоgenes (Brazova et al., 2006). In this study the authors made conjecture that reduced TGFβ1 production can contribute to cessation of endotoxin tolerance status that might lead to “cepacia syndrome” development. In our study more than two-thirds of patients from Bcc-free group had chronic colonization with *P. aeruginosa*, a pathogen that seems to induce increased TGFβ1 blood (plasma, PBL) and sputum (BAL) levels (Harris et al., 2011; Kramer and Clancy, 2018). Thus, observed TGFβ1 decrease in Bcc group may be partly due to the cytokine profile peculiarities in individuals with chronic *P. aeruginosa* infection.

In this study, for the first time, we have compared clinical characteristics and laboratory biomarkers of Bcc infected and Bcc free CF paediatric patients. Both groups had sufficient number of patients to perform statistical analyses of the data obtained. Bcc infected subjects showed a significantly increased rate of glucose metabolism disturbances and a survival disadvantage during prolong follow-up period. Biomarkers analyses revealed elevated TNF-α and decreased IL-17F levels in the sputum samples obtained from patients with Bcc infection. These patients also demonstrated the signs of reduction in systemic inflammatory response, such as decreased plasma pro-inflammatory (IL-17F and IL-18) cytokine concentrations and improvement of peripheral blood lymphocyte sensitivity to steroid treatment. Reduction in IL-17F levels may have several important consequences including increase in steroid sensitivity and glycemic control disturbances. Unfortunately, our study was not designed to determine the role of IL-17F deficiency in CF complication development. Further investigations are needed to assess the role of IL-17 cytokines in CF pathogenesis. Low plasma TGFβ1 and IL-10 levels in Bcc infected group may be a sign of subverted activity of regulatory T cells. Such immune alterations may be one of the factors contributing to the development of the *cepacia* syndrome.

## Data availability statement

The raw data supporting the conclusions of this article will be made available by the authors, without undue reservation.

## Ethics statement

The studies involving humans were approved by Ethics Committee of the Research Centre for Medical Genetics, Moscow, Russia. The studies were conducted in accordance with the local legislation and institutional requirements. Written informed consent for participation in this study was provided by the participants’ legal guardians/next of kin.

## Author contributions

GS: Conceptualization, Data curation, Formal analysis, Funding acquisition, Investigation, Methodology, Project administration, Resources, Software, Supervision, Validation, Visualization, Writing – original draft, Writing – review & editing. DP: Data curation, Investigation, Resources, Visualization, Writing – review & editing. VS: Data curation, Investigation, Resources, Visualization, Writing – original draft. SS: Conceptualization, Data curation, Methodology, Validation, Writing – review & editing. LA: Data curation, Investigation, Resources, Software, Validation, Writing – review & editing. StK: Data curation, Methodology, Validation, Writing – review & editing. AG: Data curation, Investigation, Resources, Writing – review & editing. SvK: Funding acquisition, Resources, Supervision, Writing – review & editing. RZ: Funding acquisition, Supervision, Writing – review & editing. NK: Conceptualization, Methodology, Project administration, Supervision, Writing – review & editing.

## Supplementary material

Supplementary Table 1 Characteristics of the patients who died during cross-sectional period of the study. * – the data at the moment of death.

Supplementary Table 2 PHA-induced proliferation and steroid sensitivity of peripheral blood lymphocytes in Bcc free and Bcc infected patients. Data are presented as median values (minimum values ÷; maximum values). The results were analyzed using the Mann-Whitney U test.

Supplementary Table 3 Mortality rate and survival time of Bcc infected/free patients. The data (excepted mortality rate) are shown as the mean ± standard error of the mean; the differences between the groups were assessed using Mann-Whitney U Test. * - the data were analyzed using Fisher exact test.

Supplementary Table 4 Age of Bcc establishing and colonization longevity in survived and died patients.

Supplementary Figure 1 Diversity of *Burkholderia cepacia* complex bacteria.
