# Supplementary material for: Burkholderia cepacia in cystic fibrosis children and adolescents: overall survival and immune alterations
Source: Front Cell Infect Microbiol. 2024 Jul 1;14:1374318. doi: 10.3389/fcimb.2024.1374318 (PMC11246859; doi:10.3389/fcimb.2024.1374318)
Supplement: Supplementary Table 1 — Characteristics of the patients who died during cross-sectional period of the study. * – the data at the moment of death. [file Table_1.docx]

Supplementary Table 1. Characteristics of the patients who died during cross-sectional period of the study

| **№** | **Age*, years** | **Sex** | **CFTR mutations** | **FVC, %** | **FEV_1_, %** | **BMI, kg/m^2^** | **Bcc colonization longevity*, years** | **Chronic colonization** | **CF complications** | **Prednisolone therapy** |
| --- | --- | --- | --- | --- | --- | --- | --- | --- | --- | --- |
| ***Patients with chronic Bcc infection*** | | | | | | | | | | |
| 1 | 8 | Female | F508del/CFTRdele2,3 | 51 | 40 | 12.6 | 3.5 | *P.aer.* | CFRD | + |
| 2 | 17 | Female | F508del/2143delT | 52 | 25 | 14.7 | 4.5 | — | — | — |
| 3 | 12 | Male | F508del/F508del | 106 | 115 | 13.9 | 5.0 | *St.aur.* | Cirrhosis with portal hypertension | — |
| 4 | 17 | Female | F508del/F508del | 39 | 32 | 12.0 | 6.0 | *St.aur.* | CFRD | + |
| 5 | 19 | Female | F508del/F508del | 43 | 36 | 14.4 | 7.0 | *P.aer.* | CFRD | — |
| 6 | 18 | Male | F508del/F508del | 42 | 20 | 15.6 | 6.0 | *—* | CFRD | + |
| 7 | 17 | Female | F508del/F508del | 78 | 50 | 14.7 | 5.5 | — | CFRD | — |
| 8 | 10 | Male | F508del/? | 30 | 19 | 11.4 | 1.5 | *St.aur.* | Glucose intolerance | + |
| 9 | 13 | Male | CFTRdele2,3/ G542Х | 125 | 85 | 15.6 | 4.0 | — | Cirrhosis with portal hypertension | — |
| M±m | 14.5±1.3 |  |  | 62.9±11.0 | 46.9±10,8 | 13.9±0.5 | 4.8±0.5 |  |  |  |
| ***Patients without Bcc infection*** | | | | | | | | | | |
| 1 | 16 | Male | G542X/? | 29 | 22 | 12.2 | — | *P.aer.*, *St.aur.* | — | + |
| 2 | 15 | Female | ND | 18 | 28 | 11.8 | — | *Achrom.xyl.* | — | + |
| 3 | 9 | Male | F508del/? | 50 | 38 | 11.9 | — | *P.aer.* | — | + |
| 4 | 13 | Female | CFTRdele2,3/ F508del | 64 | 52 | 14.5 | — | *P.aer.* | CFRD, cirrhosis with portal hypertension | + |
| 5 | 8 | Female | ND | 133 | 86 | 16.0 | — | *P.aer.*, *St.aur.* | CFRD | + |
| 6 | 8 | Female | F508del/ ? | 69 | 59 | 13.0 | — | *P.aer.*, *St.aur.* | — | + |
| 7 | 8 | Female | F508del/ ? | 80 | 62 | 10.5 | — | *P.aer.* | — | + |
| 8 | 15 | Female | F508del/2143delT | 38 | 30 | 14.2 | — | *P.aer.* | — | + |
| M±m | 11.5±1.3 |  |  | 60.1±12.8 | 47.1±7.6 | 13.0±0.6 | — |  |  |  |
| p | *0.0922* |  |  | 0.8099 | 0.5966 | 0.3123 |  |  | **0.0134** | **0.0204** |

* – the data at the moment of death.
