# Supplementary material for: Burkholderia cepacia in cystic fibrosis children and adolescents: overall survival and immune alterations
Source: Front Cell Infect Microbiol. 2024 Jul 1;14:1374318. doi: 10.3389/fcimb.2024.1374318 (PMC11246859; doi:10.3389/fcimb.2024.1374318)
Supplement: Supplementary Table 2 — PHA-induced proliferation and steroid sensitivity of peripheral blood lymphocytes in Bcc free and Bcc infected patients. Data are presented as median values (minimum values ÷; maximum values). The results were analyzed using the Mann-Whitney U test. [file Table_2.docx]

**Supplementary Table 2**. **PHA-induced proliferation and steroid sensitivity of peripheral blood lymphocytes in Bcc free and Bcc infected patients**

| Parameters | CF patients | |  |
| --- | --- | --- | --- |
|  | with Bcc | without Bcc | p |
| Proliferative response (count per min) | 71451  39345 ÷ 93671 | 54704  14529 ÷ 109798 | *0.806* |
| ED50 (μM) | 14.7  4.7 ÷ 112.2 | 35.8  12.2 ÷ 203.0 | *0.183* |
| **Δh (abs. units)** | **0.61**  **0.20 ÷ 1.33** | **1.13**  **0.61 ÷ 1.90** | ***0.043*** |

Data are presented as median values (minimum values ÷ maximum values). The results were analyzed using the Mann-Whitney U test.
