# Supplementary material for: Burkholderia cepacia in cystic fibrosis children and adolescents: overall survival and immune alterations
Source: Front Cell Infect Microbiol. 2024 Jul 1;14:1374318. doi: 10.3389/fcimb.2024.1374318 (PMC11246859; doi:10.3389/fcimb.2024.1374318)
Supplement: Supplementary Table 3 — Mortality rate and survival time of Bcc infected/free patients. The data (excepted mortality rate) are shown as the mean ± standard error of the mean; the differences between the groups were assessed using Mann-Whitney U Test. * - the data were analyzed using Fisher exact test. [file Table_3.docx]

**Supplementary Table 3. Mortality rate and survival time of Bcc infected/free patients**

|  | CF patients | |  |
| --- | --- | --- | --- |
|  | with *B. cepacia* | without *B. cepacia* | p |
| Mortality rate* | 25/47 (53.2 %) | 21/69 (30.4 %) | ***0.0200**** |
| Survival time (Follow-up period; months) | 68.2±4.6  59.1 ÷ 77.3 | 78.8±3.5  71.9 ÷ 85.6 | ***0.0200*** |
| Survival time (Age; years) | 19.6±0.6  18.3 ÷ 20.8 | 22.0±0.7  20.6 ÷ 23.4 | ***0.0426*** |

The data (excepted mortality rate) are shown as the mean±standard error of the mean; the differences between the groups were assessed using Mann-Whitney U Test. * - the data were analyzed using Fisher exact test.
