# Supplementary material for: Burkholderia cepacia in cystic fibrosis children and adolescents: overall survival and immune alterations
Source: Front Cell Infect Microbiol. 2024 Jul 1;14:1374318. doi: 10.3389/fcimb.2024.1374318 (PMC11246859; doi:10.3389/fcimb.2024.1374318)
Supplement: Supplementary Table 4 — Age of Bcc establishing and colonization longevity in survived and died patients. [file Table_4.docx]

**Supplementary Table 4**. **Age of Bcc establishing and colonization longevity in survived and died patients**

|  | CF patients with Bcc infection | | | |
| --- | --- | --- | --- | --- |
|  | Survived (n=25) | Dead (n=22) | p | Survived + Dead |
| Age of Bcc establishing (years) | **7.5**  **3.0 ÷ 13.0** | **10.0**  **3.0 ÷ 15.0** | **0.0498** | 9.0  3.0 ÷ 15.0 |
| Colonization longevity at the moment of biomarkers assessment (years) | 5.0  1.5 ÷ 7.0 | 4.0  1.0 ÷ 8.0 | 0.4555 | 4.5  1.0 ÷ 8.0 |
| Colonization longevity at the moment of the death or outcome assessment (years) | **11.5**  **7.0 ÷ 13.0** | **7.0**  **1.5 ÷ 14.0** | **<0.0001** | 10.0  1.5 ÷ 14.0 |
