# Supplementary figures and images for: Burkholderia cepacia in cystic fibrosis children and adolescents: overall survival and immune alterations
Source: Front Cell Infect Microbiol. 2024 Jul 1;14:1374318. doi: 10.3389/fcimb.2024.1374318 (PMC11246859; doi:10.3389/fcimb.2024.1374318)

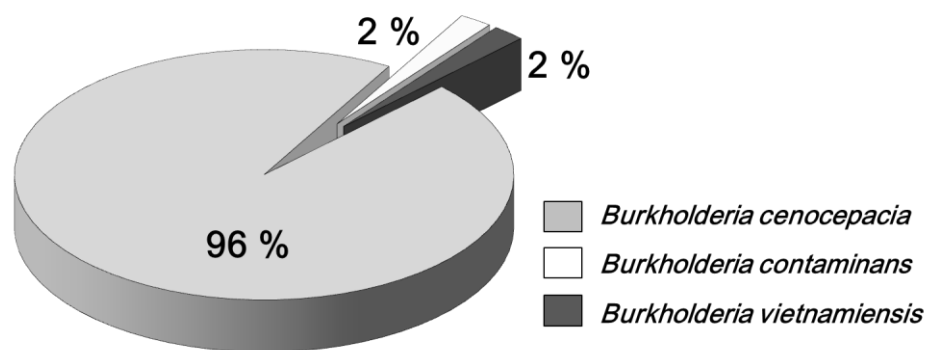

**Supplementary Figure 1. Diversity of *Burkholderia cepacia* complex bacteria**

Supplement: Supplementary Figure 1 — Diversity of Burkholderia cepacia complex bacteria. [file Image_1.pdf]
